# A New Approach with Combined Microneedle and Sublative Fractional Radiofrequency for Photoaging Management: A Clinical, Histometric, and Immunohistochemical Study

**DOI:** 10.1007/s00266-024-04416-0

**Published:** 2024-10-16

**Authors:** Moetaz El-Domyati, Osama Moawad, Hossam Abdel-Wahab, Ezzeldin F. Behairy, Ahmed F. Rezk

**Affiliations:** 1 Department of Dermatology, Faculty of Medicine, Minia University, Minia, Egypt; 2 Moawad Skin Institute for Laser, Cairo, Egypt

**Keywords:** Photoaging, Fractional radiofrequency, Microneedling, Sublative radiofrequency

## Abstract

**Background:**

Fractional radiofrequency (FRF) has been reported to be effective in improving wrinkles. A combination of microneedle and sublative fractional radiofrequency (SFRF) may have the potential synergy to improve photoaged skin.

**Objectives:**

To evaluate the efficacy and safety of combined microneedle and SFRF in photoaging management.

**Methods:**

This prospective study included 12 subjects with moderate photoaging (skin phototype III–IV). The subjects received three consecutive combined microneedle and sublative FRF at 1-month intervals. Punch biopsies were obtained before and after three months of treatment. Routine H&E, Masson-trichrome, Orcein staining, histometric measurements, as well as Collagen type I and Elastin immunohistochemical staining were performed.

**Results:**

Significant improvement was noticed regarding skin tightening and texture (*p *< 0.05), rhytides, and volunteers’ satisfaction (*p *< 0.001). Collagen fibers appeared better organized, with a significant increase in collagen type I (*p *= 0.001). Meanwhile, normal-appearing elastic fibers were restored, and a significant reduction in abnormal elastin was achieved (*p *= 0.0005).

**Conclusion:**

Combined microneedle and sublative FRF may provide a new therapeutic approach for photoaged skin.

**Level of Evidence II:**

For a full description of these Evidence-Based Medicine ratings, please refer to the Table of Contents or the online Instructions to Authors www.springer.com/00266.

## Introduction

Photoaging comprises the clinical and histopathological effects following chronic exposure to Ultraviolet Radiation (UVR) [1]. Fine and coarse rhytides, xerosis, sallowness, roughness, loss of tone and resiliency, uneven pigmentation, and telangiectasias are all clinical manifestations of photoaged skin. Histologically, collagen is broken down, and malfunctioning glycosaminoglycans (GAGs) and proteoglycans are visible in the dermis. The distinctive feature is the buildup of abnormal elastotic material, which causes a yellow tint [2].

Currently, many treatments for the symptoms of photoaging are designed to stimulate collagenogenesis. Skin resurfacing allows better organized and newer dermal matrix and epidermal normalization. Thus, for effective resurfacing, regeneration of the epidermis and portions of the dermis is required, thereby improving the appearance and health of the aged skin [3]. As the epidermis is spared, non-ablative resurfacing offers a safer, yet modest net effect, with a shorter downtime, than ablative approaches [4].

More recently, an intermediate approach using fractional energy delivery has been developed to overcome the limitations of both ablative and non-ablative treatments. In fractional resurfacing, thermally ablated epidermal and dermal microscopic zones are spaced in a grid over the skin surface; the nonablated zones in the uninjured surrounding tissue serve as a reservoir of cells that accelerate and promote safe and rapid healing. Overall, this technique increases efficacy compared to non-ablative resurfacing and has faster recovery than ablative resurfacing [5].

Radiofrequency (RF) devices use electromagnetic radiation to conduct alternating electric current to biological tissues, causing the motion of charged particles against the tissue’s resistance (impedance). This kinetic energy is converted to thermal energy. The heat, due to the flow of electrons in an electromagnetic field through tissue impedance, results in a larger coagulative dermal zone in comparison to the epidermal ablative area. In contrast to laser, RF is chromophore independent and has a better safety profile for all skin types regarding pigmentary changes [6, 7].

Various RF devices are available including monopolar, bipolar, fractional, and multipolar energy modes, which typically contain a generator and a handheld applicator with electrodes [8]. In 2008, the FDA approved fractional radiofrequency (FRF) to offer skin rejuvenation; this bipolar RF system generates fractional zones of electrothermal damage deep in the dermis, surrounded by areas of undamaged tissue under a more-or-less intact epidermis. These areas of damage in the dermal matrix induced a vigorous wound-healing process supported by the surrounding undamaged tissue and led to the synthesis of new collagen and elastin, dermal remodeling, and replenishment of hyaluronic acid [9].

FRF appears to acquiesce to the pros of the ablative and non-ablative laser therapy while being associated with fewer side effects; making it an attractive choice by blending both benefits [10]. Brightman et al. (2009) introduced the term “sublative” as a derivative of “sub-ablative,” which described the ability to ablate the superficial epidermis together with the generation of pyramid-shaped thermal energy underneath the ablated epidermal zone [11], unlike the columnar shape of ablative lasers. It is accepted that the effective impact of a bipolar RF electrode is half the distance between the electrodes. Therefore, the 64-pin-electrode, having an inter-pin distance of 1.5 mm, may impact the tissue up to 750 μm [12, 13].

More recently, the technology has been combined with nonenergy-based devices such as microneedling to improve skin rejuvenating outcomes by enhancing dermal, subdermal, and adipose heating while minimizing epidermal heating and its complications. Multiple studies have shown the safety and efficacy of RF microneedling in facial aesthetics, including skin rejuvenation, acne scars, and melasma. The most common side effects are pain, erythema, purpura, and edema, which resolve on average within few days [14].

Microneedle fractional radiofrequency (MFRF) has recently gained popularity as a promising strategy combining effectiveness and safety in skin rejuvenation. This method produces targeted cutaneous injury with a minimum amount of superficial involvement using microneedles or electrode pins that are minimally invasive. Collagen fibrils become denaturated because of the heat insult, which starts the healing process [15]. As the thermal effect of RF devices is related to the impedance and conductivity of the skin, the energy impact around the microneedle electrode is narrower at the epidermal surface but broader in the dermis, in contrast to previous laser-based fractional systems [16]. Needles can also be insulated or non-insulated. Theoretically, insulated needles minimize heat dissipation at the epidermal layer and may be more likely to protect against epidermal injury than non-insulated needles [14].

Since sublative fractional radiofrequency (SFRF) can improve superficial irregularities with short downtime, and MFRF with insulated microneedles can improve the status of the dermal matrix via precise deep dermal damage; combining these two modalities might produce synergistic efficacy in the management of photoaged skin. Therefore, the current study aims to evaluate the efficacy of the new combined approach of MFRF and SFRF in managing photoaged skin, through subjective clinical assessment, together with objective histological and immunohistochemical assessment.

## Materials and Methods

### Study Population

This prospective study involved 12 volunteers seeking to improve facial skin laxity and wrinkles. Recruitment took place at the dermatology outpatient clinic. These volunteers had Fitzpatrick skin types III (30%) and IV (70%) and exhibited classes I–II wrinkles on the Glogau scale [17].

The study included individuals with sun-induced photoaged facial skin. Exclusion criteria were pregnancy, lactation, any signs of skin infection or inflammatory disease, history of hypertrophic scars or keloids, use of oral isotretinoin in the past year, or previous facial rejuvenation procedures.

Two dermatologists assessed the improvement percentage based on the Quartile Grading Scale, side effects, and volunteers’ satisfaction. The degree of improvement was classified as very good (76–100% improvement), good (51–75%), moderate (26–50%), mild (1–25%), and nil (0% improvement). The study received approval from the Ethical Research and Scientific Committee, and all volunteers were informed about the treatment’s benefits, risks, and potential complications before providing consent.

### Device Description

We used a Dermatrix Duo device (Shenzhen GSD Tech Co., Ltd., China) with dual-mode fractional RF at 1.15 MHz. It has an MFRF handpiece with 49 insulated-coated needles for RF delivery to an 8x8 mm dermis area. The microneedle has a width of 0.2 mm, with a non-insulated tip measuring 0.5 mm in length and 0.03 mm in diameter. The SFRF handpiece utilizes needle-free dot matrix RF technology with 64 multi-electrode pins (21x21 mm).

### Treatment Regimen and Follow-Up

After a gentle cleanse with 70% isopropyl alcohol, EMLA cream (2.5% Lidocaine + 2.5% Prilocaine) was applied generously for 45-60 minutes. Prior to treatment, the cream was removed, and the area was disinfected with 10% povidone-iodine and alcohol. A white cosmetic pencil outlined the target area. For the MFRF handpiece, settings were: energy 6–12J, depth 0.5–1.0mm, pulse width 50–120ms, and rest time 200–600ms. The SFRF handpiece was set to: energy 2–4J, pulse duration 100-150ms, and penetration depth M3–M4 (ranges from superficial (M1) to deep (M4)).

The skin was gently pinched to reduce pain and bleeding. The handpiece was applied firmly and perpendicular to the treatment area, with RF energy delivered via footswitch. Each cosmetic unit underwent three passes: the first at 1 mm depth with MFRF handpiece, the second with less energy at 0.5 mm depth, and the third with SFRF handpiece. A 25% overlap was allowed. Optimal results were achieved through three sessions, spaced 4 weeks apart.

Cold compresses were applied immediately after each session to alleviate pain and inflammation. Patients were advised to use antibiotic ointment for three days and refrain from using other topicals like sunscreen or makeup until complete skin healing (typically 3–5 days). Before-and-after photos were taken three months apart. Punch biopsies (3 mm) were taken from the face before treatment and one month after the final session, from a site near the initial biopsies.

### Histological, Histometric and Immunohistochemical Staining

Tissues were fixed in 10% buffered formalin, embedded in paraffin, and sectioned into 5 µm -thick sections. Standard hematoxylin-eosin (H & E), Orcein (elastic fibers) (O7505; Sigma Aldrich, St. Louis, Missouri, USA), and Masson Trichrome stains (collagen) (HT15; Sigma Aldrich, St. Louis, Missouri, USA) were performed. Meanwhile immunoperoxidase (IP) technique was performed to evaluate collagen type I and total elastin. Histometric measurements were performed on H&E-stained sections, at the dermatopathology unit, using a computer assisted program with installed analySIS^®^ Five software (Olympus Soft Imaging Solutions GmbH, Johann-Krane-Weg 39, D-48149 Münster, Germany).

For immunohistochemical staining, slides were heated at 60 °C for 30–60 minutes, and then deparaffinized and antigens were retrieved in 0.1M sodium citrate (pH 6.0) for 5 min by the microwave method. Nonspecific sites were blocked, and tissues were incubated with antibodies to collagen type I (1:400; sc-59772; Santa Cruz Biotechnology) and total elastin (1:300; E4013; Sigma Aldrich). Samples were then incubated with biotinylated secondary antibody (1:200; PK-6102; Vector labs, Burlingame, California), ABC reagent (Vectastain Elite ABC Peroxidase Kits Mouse; PK-6102; Vector labs) and stained with DAB Chromogen Substrate Kit (K3468; Dako, CA, USA). All slides were counterstained with hematoxylin (7211; Thermo Scientific) and mounted for viewing. Quantitative evaluation of immune-stained tissues was carried out using computer-based analySIS^®^ Five software (Olympus Soft Imaging Solutions GmbH, Johann-Krane-Weg 39, D-48149 Münster, Germany).

### Statistical Analysis

Data were analyzed using the software package for statistical science (SPSS for Windows, Version 16, SPSS Inc, Chicago, IL). Statistical analysis was performed using the Wilcoxon-matched pairs signed ranks test and the Pearson x^2^ test. Data were expressed as mean ± SD. A *p *≤ 0.05 was considered statistically significant, and a *p* ≤ 0.001 was considered highly significant.

## Results

The present study was conducted on 12 patients, nine females (75%) and three males (25%), attending the outpatient dermatology clinic to treat facial wrinkles and laxity in photoaged skin. The age of the patients ranged from 47 to 62 years, with a mean ± SD of 55.3 ± 5.9.

### Clinical Evaluation

**Fig. 1:**
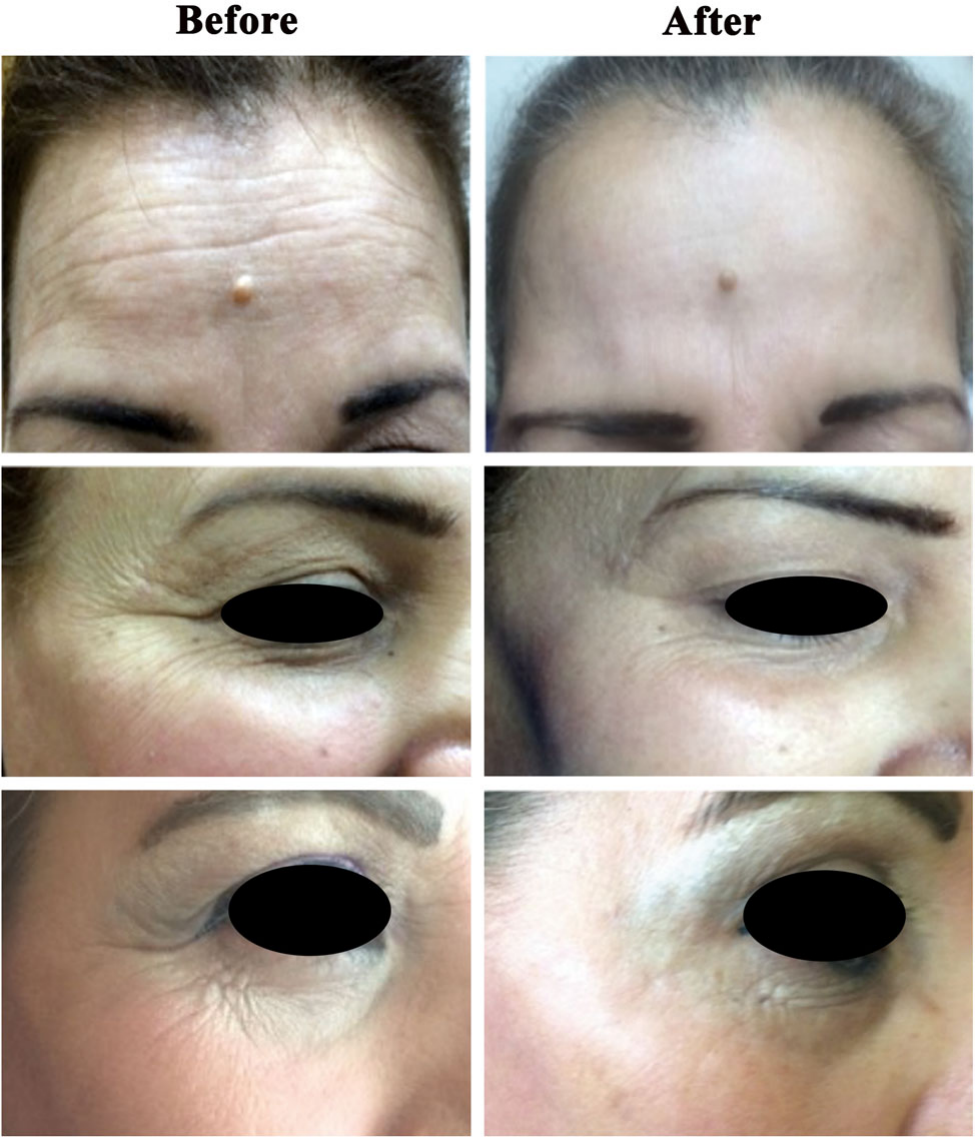
A photo of volunteers with photoaged skin showing moderate to marked improvement in skin tightening, texture, and wrinkles in the forehead as well as the crow’s feet area at the end of treatment compared to baseline

All 12 participants completed the study, displaying evident clinical improvement in skin laxity and wrinkles around the eyes and forehead (Fig. 1). Post-treatment, subjects experienced substantial improvements in skin tightening (76–82%, *p* = 0.005), skin texture (70–85%, *p* = 0.003), wrinkle reduction (85 to 96%, *p* = 0.0004), and reported high satisfaction (86–97%, *p* = 0.0002) (Table 1). Observers’ assessments aligned with volunteers’ evaluations. Common side effects included erythema, edema, burning sensation, and pruritus, varying with energy level and individual skin sensitivity. Transient epidermal crusting, resolving in 3–5 days, was also noted. Despite these effects, the benefits outweighed the drawbacks.

**Table 1:** Percent rate of clinical improvement, based on volunteers’ evaluation (n=12), following FRF treatment relative to baseline

(N = 12)	Percent improvement (%) After treatment	*p* value
Skin tightening	76–82	0.005*
Skin texture	70–85	0.003*
Rhytides	85–96	0.0004**
Overall satisfaction	86–97	0.0002**

The degree of improvement was evaluated based on the Quartile Grading Scale.

*p ≤ 0.05

**p ≤ 0.001

### Histologic and Histometric Evaluation

**Fig. 2:**
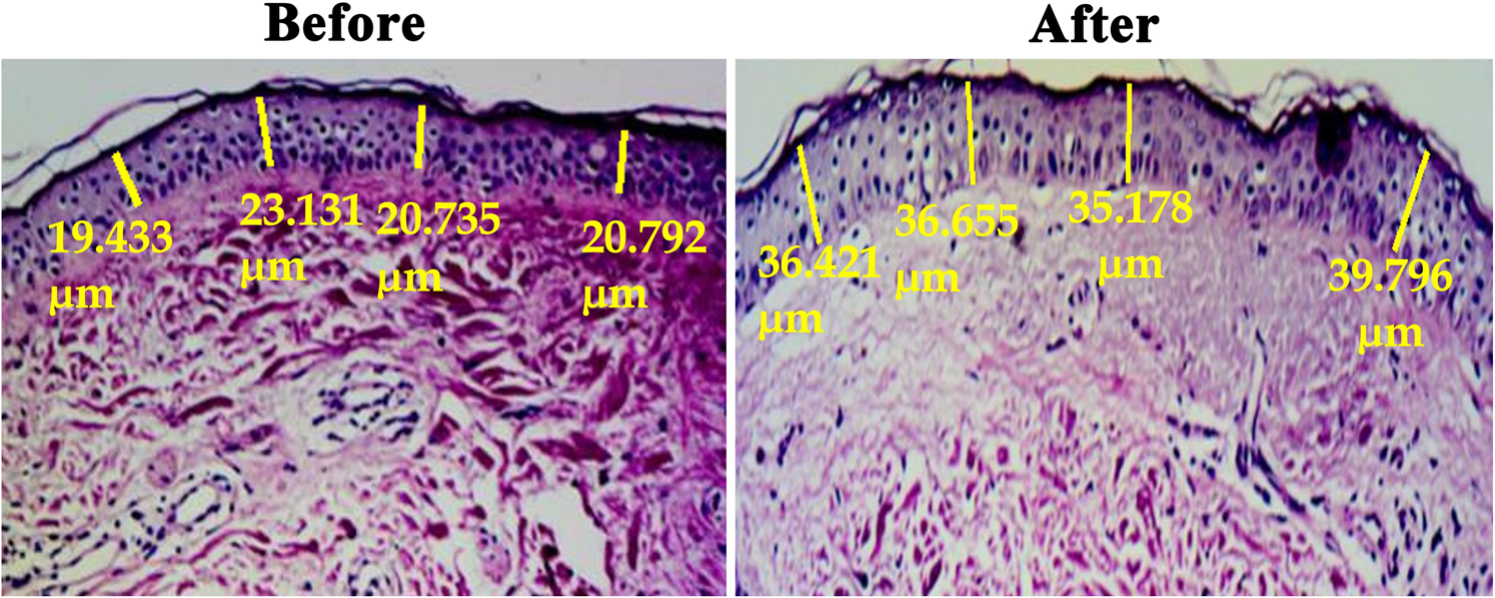
Histometric evaluation of H&E-stained sections showing increased epidermal thickness at the end of treatment compared to baseline (H&E, × 200)

Histological evaluation of skin biopsies revealed properly organized collagen fibers after treatment. Moreover, histometric evaluation revealed a significant increase in epidermal thickness following treatment (41.80 ± 4.51 µm) compared to baseline (29.53 ± 4.86 µm) (*p* < 0.0001) (Fig. 2).

### Evaluation of Collagen Changes in the Dermis

**Fig. 3:**
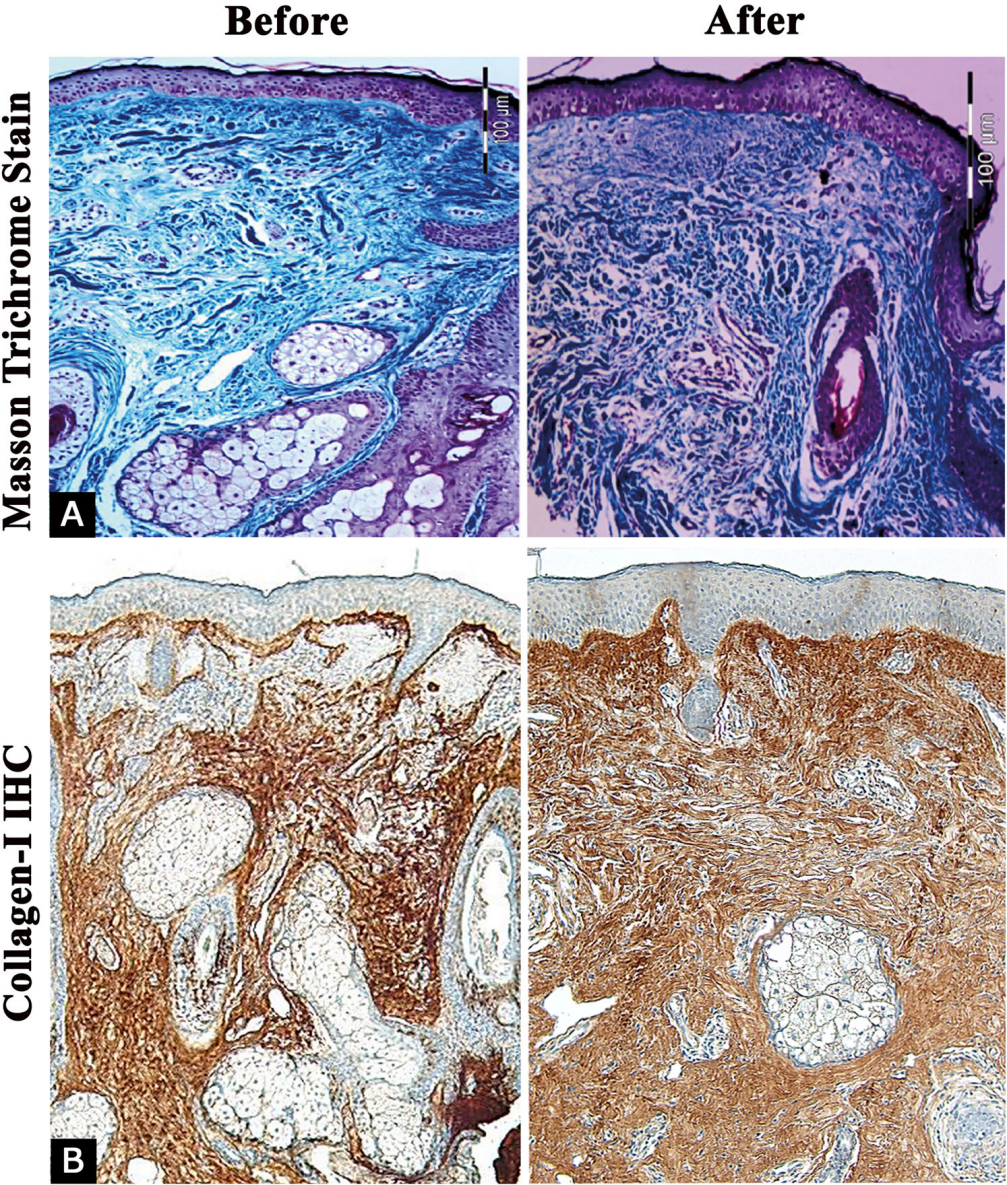
Examination of collagen fibers showing increased and well-organized collagen fibers after treatment compared to baseline using Masson-trichrome stain (**A**)**,** as well as Immunostaining of Collagen type I (**B**) (× 200)

Pre-treatment Masson Trichrome staining showed disorganized collagen bundles with increased interfibrillar spaces. Post-treatment, collagen fibers appeared more organized and compact with reduced interfibrillar spaces. Additionally, quantitative evaluation of collagen type I showed a significant increase three months after treatment (57.83 ± 4.35) compared to baseline (47.08 ±   4.27) (*p* = 0.001) (Table 2) (Fig. 3).

**Table 2:** Quantitative objective evaluation of elastin and collagen type I percent in photoaged skin before and after treatment

(N = 12)		Before	After	*p* value
Collagen I (%)	Range	40–55	50–64	0.001**
	Mean ± SD	47.08 ± 4.27	57.83 ± 4.35	
Elastin (%)	Range	16–26	13–21	0.0005**
	Mean ± SD	22.67 ± 2.81	18.25 ± 2.05	

Quantitative objective evaluation was performed using computer-based analySIS^®^ Five software.

*SD* standard deviation

**p ≤ 0.001

### Evaluation of Elastin and Elastic Fiber Changes in the Dermis

**Fig. 4:**
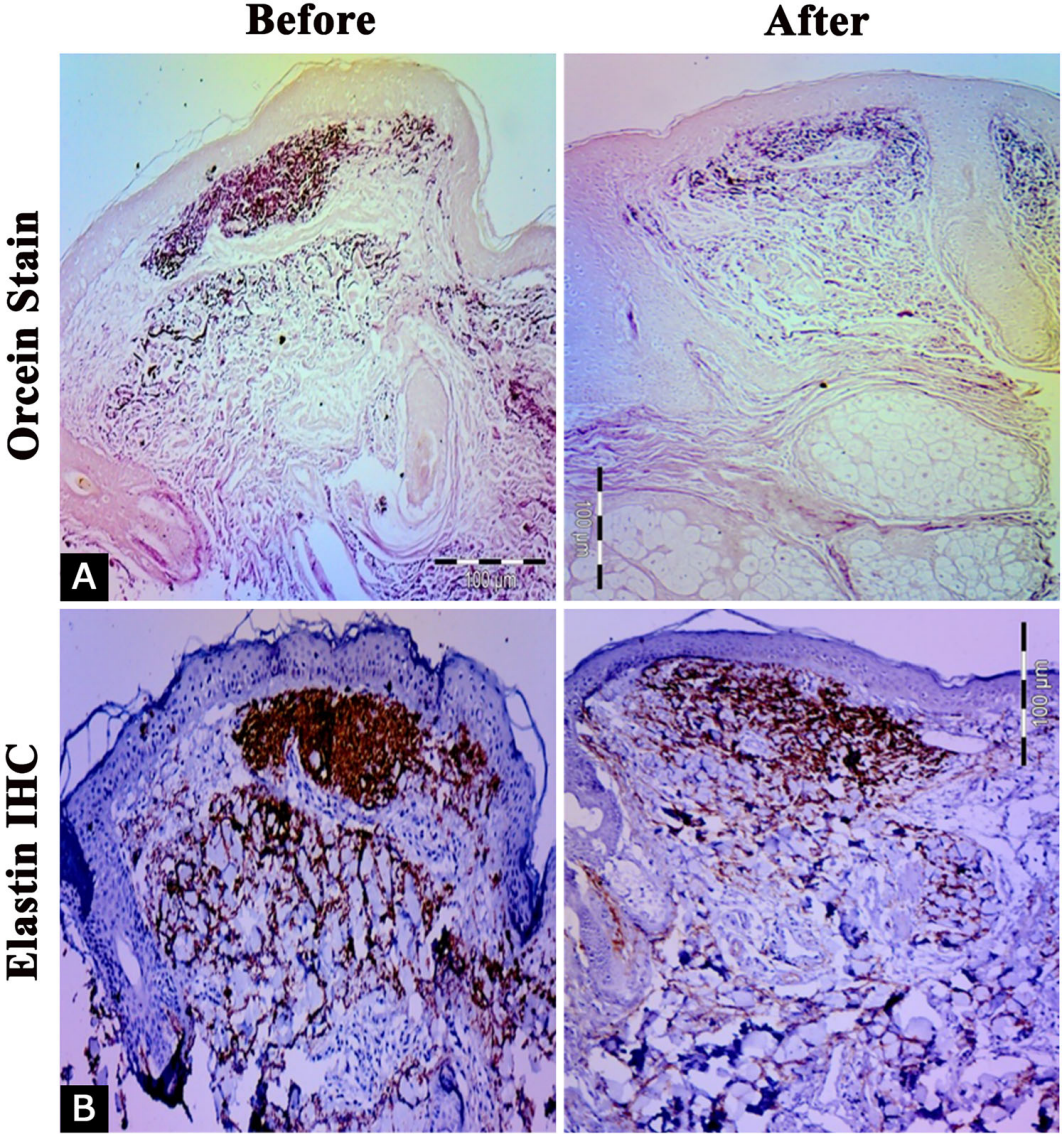
Examination of elastic fibers showing decreased solar elastosis, as well as restoration of normal-appearing elastic fibers within the papillary and upper reticular dermis after treatment compared to baseline using Orcein stain (**A**) and Immunostaining of Elastin (**B**) (× 200)

In photodamaged skin, there was notable abnormal deposition of elastic fibers in the dermis, along with an accumulation of elastin under the epidermis forming elastotic material. FRF treatment led to a significant reduction in total dermal elastin, observed three months post-treatment (18.25 ± 2.05) compared to baseline (22.67 ± 2.81) (*p* = 0.0005) (Table 2) (Fig. 4).

## Discussion

Over the past few decades, various interventional modalities have been developed to improve the appearance of photoaged skin. Traditionally, ablative laser remained the gold standard with robust efficacy but notable drawbacks and considerable downtime [5, 18, 19]. Various non-ablative procedures are receiving more attention since they can rejuvenate the skin with minimal downtime and fewer adverse effects [20–22].

In the 1990s, RF emerged in cosmetic dermatology as a minimally invasive method for improving skin aging. By applying alternating electric current, RF technology generates kinetic energy that is converted to thermal energy [23]. This process triggers collagen contraction and stimulates new collagen production, leading to dermal remodeling and skin tightening. RF is considered safe for darker skin types as it does not selectively target chromophores [22, 24].

Studies on RF microneedling for skin rejuvenation reveal a 20–60% mean improvement in facial rhytides, skin laxity, and texture roughness after one to three sessions. Noticeable effects start at one month, peak at three months, and last up to seven months. Additionally, RF microneedling is well-tolerated and safe for all skin types [25, 26].

Since Hantash et al.’s pioneering work on MFRF in 2009 [9, 27], numerous studies have examined its histological and cosmetic effects in both animal and human models, employing both insulated and non-insulated needles. Clinical research has shown its effectiveness in treating diverse skin issues like striae, texture irregularities, wrinkles, hyperpigmentation, skin laxity, and acne scars [28–30]. Additionally, FRF therapy has been utilized by many to counteract various signs of skin photoaging [4, 18, 31].

Many researchers, including us, attribute the superior results of ablative modalities to the induced epidermal injury. This prompts the immediate release of growth factors, cytokines, and compounds from injured blood vessels and platelets, initiating the repair process [32].

Brightman et al. (2009) coined the term “sublative” to describe the generation of a pyramid-like thermal zone beneath the ablated epidermis. Studies have shown that SFRF treatments with a superficial tip led to effective epidermal regeneration and remodeling in the superficial dermis [11]. RF is typically most effective for individuals with mild to moderate signs of aging, hence studies often include participants in the Glogau I-II range of photoaging [33, 34].

The present study introduced a novel FRF technique, combining MFRF followed by SFRF. This approach synergistically targeted multiple skin layers, from epidermis down to dermis, resulting in significant improvement for over 70% of subjects.

The lack of standardized methods for measuring wrinkles and skin elasticity is a fundamental issue in rejuvenation studies. Clinical outcomes often rely on subjective observations, which can be inconsistent. Additionally, photo-documentation may not adequately capture therapy quality and efficacy [35, 36].

In this study, we aimed to enhance accuracy by employing objective measures to evaluate FRF’s effectiveness on photoaged skin. This involved precise histometric, histochemical, and immunostaining techniques before and after treatment. We found notable increases in epidermal thickness and collagen type I, along with a significant reduction in abnormal elastin three months post-treatment.

For a long time, the role of adipose tissue in skin aging was not fully elucidated. Traditionally, anti-aging treatments have focused primarily on dermal fibroblasts and collagen. However, recent research has highlighted the significant roles of both sWAT (subcutaneous white adipose tissue) and dWAT (dermal white adipose tissue) in the aging process [37].

Dermal white adipose tissue is located among collagen bundles in the reticular dermis, near blood vessels and hair follicles [38]. It can form clusters around pilosebaceous units or be diffuse throughout the dermis [39]. Dermal white adipose tissue secretes adipokines that influence fibroblasts and endothelial cells, affecting collagen synthesis and angiogenesis [38, 40]. It also supports skin repair, elasticity, and firmness [39, 41]. Meanwhile, radiofrequency electromagnetic fields can influence adipose tissue thermoregulation and mitochondrial signaling [42].

Our protocol integrates the stimulation of epidermal healing through SFRF and dermal healing through MFRF, acknowledging the importance of dWAT. By administering MFRF followed by SFRF in sequence, we have achieved remarkable results. The controlled heat application at a moderate depth is also designed to stimulate the release of growth factors from dWAT and adipose-derived stem cells (ADSCs), which enhances tissue repair. This approach is expected to accelerate wound healing and reduce recovery time. Most subjects experienced significant improvements, both subjectively and objectively. This innovative method benefits from targeting distinct skin layers and activating fibroblasts, dWAT, and ADSCs, ultimately restoring skin integrity and function.

Patients experienced a slightly longer downtime (3–5 days) compared to MFRF alone, but the excellent results justified the extended recovery. The study’s limitation lies in the absence of a control group, making it uncertain to attribute synergistic benefits to the combined treatment. Future research should focus on optimizing parameters like energy levels, needle depth, session frequency, and total sessions in a larger sample size over an extended period for more conclusive evidence on the effectiveness of this combination RF therapy, comparing it to MFRF monotherapy with insulated and non-insulated microneedles.

In conclusion, combining MFRF and SFRF produced excellent results in treating photoaged skin, with short-lived and manageable side effects. It’s a safe, well-tolerated, and effective therapy for rejuvenating photoaged skin. While the treatment appeared safe in this small sample, further research with a larger cohort is necessary to fully assess the safety profile of such treatment, and also to delve into the significance of different forms of electromagnetic-induced skin damage and repair mechanisms.
